# Delphi method consensus on radiographic characteristics influencing management decisions for proximal humerus fracture

**DOI:** 10.1186/s13018-025-06465-w

**Published:** 2025-11-26

**Authors:** Sarah B. Floyd, Maria Cordero Romero, Charles Daly, Stephan G. Pill, Michael Kissenberth, Kyle Jeray

**Affiliations:** 1 Department of Public Health Sciences, Clemson University, 201 Epsilon Zeta Drive, Clemson, SC 29634 USA; 2 Department of Orthopaedics and Physical Medicine, Medical University of South Carolina, 268 Calhoun St, Charleston, SC 29425 USA; 3 Steadman Hawkins Clinic of the Carolinas, Prisma Health, 200 Patewood Dr, Suite C100, Greenville, SC 29615 USA; 4 Department of Orthopaedic Surgery, Prisma Health, 701 Grove Rd, Greenville, SC 29605 USA; 5 Novant Health, Orthopaedics and Sports Medicine, South Carolina 29615 Greenville, USA; 6 University of South Carolina, School of Medicine Greenville, South Carolina 29605 Greenville, USA

**Keywords:** Proximal humerus fractures, Delphi method, Consensus, Shoulder, Classification, Fracture features

## Abstract

**Background:**

An individualized, and evidence-based approach is needed for effective management of proximal humerus fractures (PHF). As a first step toward this goal, expert agreement is needed on the fracture features that are relevant to management decisions. In this study, we utilize an e-Delphi method to obtain consensus on a comprehensive list of fracture features important to PHF management.

**Methods:**

A literature review was conducted to identify all PHF features of importance. A panel of trauma and shoulder surgeons with expertise in treating PHF were recruited. The e-Delphi method consisted of two rounds of 4-point Likert scale questions. In the first-round participating surgeons were asked to assess each feature’s level of importance on management decisions, and had the option of proposing modifications to current feature definitions and recommending new features for inclusion in Round 2. Final consensus was reached for a fracture feature if at least 78% of surgeons indicated it was “Very Important” or “Important” in Round 2. The 78% consensus threshold was chosen based on prior item content validity threshold recommendations.

**Results:**

The e-Delphi participants consisted of 29 physicians which completed two rounds of surveys. Round 1 began with 10 features and two additional features, bone quality and humeral head subluxation, were recommended by participants. Of a total of 12 features, 8 reached consensus in Round 2 which included: dislocation (100%), head split fracture (100%), topographical parts (97%), head shaft translation (93%), displacement of parts (90%), bone quality (83%), head impaction (83%), and head shaft angulation (83%).

**Conclusions:**

Consistent with prior research, we identified dislocation, head split fracture, topographical parts, and displacement of parts as important features guiding management decisions for PHF. Our results also distinguish head shaft angulation, head shaft translation, head impaction, and bone quality as key PHF features that are relevant when making treatment decisions. We anticipate that these results can provide a foundation for future research efforts to identify patient subgroups and evaluate comparative treatment outcomes for PHF.

**Supplementary Information:**

The online version contains supplementary material available at 10.1186/s13018-025-06465-w.

## Background

Proximal humerus fractures (PHF) are the third most common fracture in individuals aged 65 and older [1] with incidence increasing in this population [2]. Despite the high prevalence of PHF injuries, the optimal treatment approach for patients with PHF remains unclear [3–5]. Many studies have attempted to provide evidence to guide initial decisions surrounding operative or non-operative management [6]. There have been 11 RCTs [7–18] and 11 meta-analyses of RCTs [19–29] comparing operative and non-operative management of PHF over the past 4 decades. Nevertheless, methodological limitations of these studies including different study populations, exclusion criteria, fracture types, patient ages, and treatment comparators have limited our ability to summarize findings into consistent conclusions on how individual patients should be treated [30]. Optimal initial management of these injuries is crucial to limit the period of pain and disability for patients, with multiple studies correlating late and revision surgery with worse outcomes [31, 32]. Furthermore, optimal initial management decisions reduce the likelihood of fracture sequelae including non-union, significant malunion, tuberosity resorption, loss of rotator cuff function, post-traumatic arthrosis, and persistent subluxation or dislocation which can perpetuate as long-term complications [33–37].

Historically, the choice of initial treatment has been guided by a balanced consideration of both patient-specific factors including age, overall health, and lifestyle goals, in addition to the morphological fracture characteristics [42, 43]. To date, 22 different PHF classification systems have been developed [42, 44, 47–66], signaling that our knowledge of important morphological fracture features continues to expand. Yet, we have failed to establish a classification system that is evidence-based, well agreed upon, and easily implemented in clinical practice [52]. The growing list of potentially important fracture features, number of diverse classification systems, and poor agreement in applying fracture classification have further complicated our ability to harmonize study findings [52, 67] and contributes to the ever-growing challenges associated with the management of PHF. A next step in advancing care for patients with PHF are large-scale observational studies that consistently measure and assess the relationship between combined patient and complex fracture presentations and both treatment choice and outcomes.

The Delphi method is an established consensus methodology for obtaining expert agreement on a topic [68] and it has successfully been used in orthopaedic applications to reach agreement on best practices and guidelines [70, 71]. To date no study has been conducted to gain consensus on the comprehensive list of PHF radiographic characteristics that are relevant to treatment choice and outcomes. The objective of this Delphi study is to identify the complete list of fracture characteristics that are relevant to management decisions for PHF. In future studies, the measurement of all relevant fracture characteristics may reveal important relationships between fracture characteristics, treatment and outcomes. This Delphi study provides a first step toward generating evidence to improve individual treatment decision making for PHF.

## Methods

### Identification of important proximal humerus fracture features

A literature review was conducted to identify all classification systems developed for PHF. We searched Ovid MEDLINE and the Cumulative Index to Nursing and Allied Health Literature (CINAHL) databases for published PHF classification systems from the years 1946 to May 8, 2023. A total of 2,130 articles were initially identified from a database search, and 1,698 duplicates were removed. Of the remaining 432 articles, 77 articles were included based on title and abstract review. From there, 31 full articles from this list were scanned by the research team. This process resulted in the identification of 22 unique classification systems for PHF developed between 1960 and 2022.

Individual fracture features were extracted from each of the 22 classification systems and reviewed conjointly between the research and clinical team members which included the Orthopaedic Surgeon Working Group (OSWG). The OSWG is composed of four experienced orthopaedic surgeons subspecializing in trauma and shoulder surgery (KJ, CD, MK, SP), and served as the clinical steering committee for this work. Three surgeons, one trauma and two shoulder subspecialists, have been in practice for over 20 years and one shoulder subspecialist has been in practice for over 10 years. After reviewing the classification systems, a total of 21 unique PHF features were identified by the research team and presented to members of the OSWG. These 21 features were separately assessed and features with similar concepts were grouped together. For example, “number of parts” and “tuberosity involvement” were both grouped under topographical parts, and “head position” and “neck-head angulation” were both grouped under head shaft angulation. Definitions and feature examples were subsequently generated for each feature group. Each feature group was initially developed by the research team, presented to the OSWG, revised, and finally approved jointly by both research and clinical team members. This iterative process resulted in a total of 10 unique fracture features, and related definitions and examples slated for Round 1 e-Delphi inclusion. The full list of fracture features identified for inclusion in the e-Delphi and their definitions is shown in Table 1.

**Table 1:** The radiographic PHF features and definitions identified for inclusion in round 1

Fracture Feature	Feature Definition	Classification System
Topographical Parts	The location of topographical parts or segments involved in the fracture.	Neer 1970 [42], AO 1990 [44], Neer 2002 [48], Edelson 2004 [49], Hertel 2004 [50], MTM 2008 [51], AST 2013 [56], HGLS 2013 [55], Resch 2016 [59], Sumrein 2018 [60], Matsumura 2020 [63], Liu 2021 [64], Mayo-FJD 2022 [66]
Displacement of Parts	Greater than 1 cm distance between fragments (or 5 mm for tuberosities) or 45 degrees of angulation between fragments.	Drapanas 1960 [47], Neer 1970 [42], AO 1990 [44], Neer 2002 [48], Edelson 2004 [49], MTM 2008 [51], AST 2013 [56], HGLS 2013 [55], Mutch GT 2014 [57], Mutch GT Ratio 2014 [58], Sumrein 2018 [60], Matsumura 2020 [63]
Dislocation	The humeral head is not located within the glenoid cavity or socket.	Neer 1970 [42], AO 1990 [44], Neer 2002 [48], Edelson 2004 [49], MTM 2008 [51], AST 2013 [56], HGLS 2013 [55], Resch 2016 [59], Matsumura 2020 [63], Mayo-FJD 2022 [66]
Head Shaft Angulation	The head shaft angle is the angle between the humeral shaft axis and the humeral head axis as observed from the anterior-posterior view.	AO 1990 [44], Neer 2002 [48], Edelson 2004 [49], Resch 2011 [52], HGLS 2013 [55], Resch 2016 [59], Boileau 2019 [61], Russo 2020 [62], Mayo-FJD 2022 [66]
Head Shaft Translation	The amount of mediolateral displacement between the shaft and the head.	Neer 1970 [42], AO 1990 [44], Neer 2002 [48], Boileau 2019 [61]
Head Split Fracture	When the articular surface area of the humeral head cleaves into two or more parts.	Neer 1970 [42], AO 1990 [44], Neer 2002 [48], Edelson 2004 [49], Russo 2012 [54], AST 2013 [56], Matsumura 2020 [63], Russo 2020 [62], Mayo-FJD 2022 [66], Ganokroj 2022 [65]
Head Impaction	Forced contact between the head and shaft resulting in impaction and a crushing head deformity.	Neer 1970 [42], AO 1990 [44], Neer 2002 [48], Edelson 2004 [49], Resch 2011 [52], Carrerra 2012 [53], Matsumura 2020 [63], Russo 2020 [62]
Calcar Comminution	The presence of one or more intermediate fragments in the medial calcar area.	Edelson 2004 [49], Russo 2012 [54], Russo 2020 [62]
Metaphyseal Head Extension	The length of the medial calcar segment that remains attached to the head.	Hertel 2004 [50], HGLS 2013 [55]
Medial Hinge Disruption	Separation of the shaft and head causing disruption of the pivot point of the head at the level of the posteromedial fracture line.	Hertel 2004 [50], Russo 2012 [54], HGLS 2013 [55], Russo 2020 [62]

### Participants

Recruitment took place by snowball sampling from physician networks and in-person recruitment at two annual Orthopaedic conferences hosted in the Southeastern United States. Regional conferences for trauma surgeons and shoulder and elbow fellowship-trained surgeons, the two orthopaedic subspecialists that most often treat PHFs, were targeted for e-Delphi participant recruitment. Physicians were eligible to participate in our e-Delphi process if they were a practicing orthopaedic surgeon that managed at least 10 PHFs per year. A minimum number of years of experience was not a required eligibility criterion, as we valued the perspectives of newly trained physicians that might have new perspectives on PHF management based on the recent literature on this injury.

### Study design

Once eligibility was assessed, interested surgeons were invited to participate in the e-Delphi method by completing the panel recruitment survey which collected demographic information, subspecialty training, professional experience, and number of PHF treated annually. Completion of the panel recruitment survey served as surgeons’ consent to participate in the e-Delphi method, and surgeons from this panel were invited to participate. A total of 74 surgeons completed the panel recruitment survey, 63 completed Round 1 and 29 completed Round 2. Attrition across rounds is not unusual, but the final group completing Round 2 (*n* = 29) far exceeds the general Delphi methodology standard of 12 participants needed to enable consensus [74, 75]. Physicians attending either of the in-person research symposiums were given a $50 incentive for their participation.

We adopted an e-Delphi method [76] to gain consensus on the most important fracture features relevant to PHF clinical decision-making. The e-Delphi surveys were developed using an online survey software platform and consisted of an initial panel recruitment survey and 2 subsequent survey rounds. Rounds were conducted electronically and spanned 7 months from initial recruitment to Round 2 completion. The panel recruitment survey and Round 1 and Round 2 surveys are included in Additional Files 1–3. This study was reviewed and approved by the Institutional Review Board at Clemson University.

### Round 1

In the first round, panel members were asked to imagine they were looking at a set of plain radiographs for PHF taken no more than 2 weeks following an acute PHF injury. They were then presented with each fracture feature, a definition, feature examples, and asked to evaluate the importance of the feature on their treatment decision-making. Panel members ranked each feature from “Very Important” to “Not all Important” with a 4-point Likert scale and were given the option to provide an alternative definition or example(s) for each feature. At the end of Round 1, panel members were also given the option to list any PHF features they believed to be important that were not included in Round 1. Weekly email reminders were sent to respondents that had not completed Round 1. The results of Round 1 were reviewed by members of the research team and OSWG and informed the development of Round 2.

### Round 2

In line with the Delphi methodology [68], a second e-Delphi round was developed and sent via email to participants that completed Round 1. All participant suggestions from Round 1 were reviewed, and final modifications and new feature additions for Round 2 were made conjointly by members of the OSWG and the research team. Features that were excluded from further consideration included patient characteristics and non-radiographic features. At the beginning of Round 2, surgeons were presented with the results of the 4-point Likert scale from Round 1 for each PHF feature and asked to assess PHF feature importance for the original 10 PHF features using the same Likert scale from Round 1. Surgeons were presented with modifications to the feature name, definition, and feature examples of the original 10 features, in addition to any new features identified for inclusion in the e-Delphi method. Any modifications or new PHF features added to Round 2 of the e-Delphi were highlighted in red for panel members to easily identify. Weekly email reminders were sent to respondents that had not completed Round 2.

### Statistical analysis & consensus

All survey data was collected using Qualtrics Survey software 2023 (Qualtrics, Provo, UT). A number of approaches exist for determining consensus in Delphi methods, yet a majority of studies have determined consensus with a pre-defined percentage agreement [77]. Consensus definitions used in prior Delphi methods reveal the lowest acceptable consensus threshold of 50% [77]. In our work, we applied a more stringent percentage agreement criteria for consensus at 78%, using the more conservative item-level content validity index (I-CVI) following recommendations described by Polit et al. [78]. The I-CVI is a measure of percent agreement on the relevance of an item, and a threshold of 78% is recommended to determine item relevance where there are at least three respondents [78]. In line with these content validity recommendations, a PHF feature was considered to have reached consensus if the percentage of surgeons identifying the feature as “Very Important” or “Important” was at least 78%. Descriptive statistics and data analysis were performed using R Statistical Software (v4.3.1, R Core Team 2023).

## Results

### Participants

A total of 29 orthopaedic surgeons practicing in 16 states spanning the South (66%), Midwest (14%), West (14%), and Northeastern (3%) regions in the US and one Canadian province (3%) completed the e-Delphi survey rounds. Most of these surgeons were shoulder (72%) and trauma subspecialists (24%), had been in practice for at least 6 years (69%), and treated more than 20 PHF annually (62%).

### Round 1

The results of the first round reflect 9 of the 10 PHF features meeting the consensus threshold with at least 78% of surgeons ranking the feature as “Very Important” or “Important.” Topographical parts (100%), displacement of parts (100%), dislocation (98%), head split fracture (98%), head shaft angulation (83%), head shaft translation (81%), head impaction (79%), and metaphyseal head extension (79%), and calcar comminution (78%) met the consensus threshold, while medial hinge disruption (75%) did not. The results of Round 1 are shown in Table 2.

Physician respondents were given the option to offer revised feature definitions in Round 1. Displaced lesser tuberosity fractures were added to the displacement of parts feature examples. Also, more specific language acknowledging a complete translation was added to the feature example for head shaft translation. Clarifying text was also added to metaphyseal head extension, denoting the medial position of the segment of interest. In the optional free text section at the conclusion of Round 1, bone quality and humeral head subluxation were identified by several panel members as distinct radiographic features of importance to be included in the e-Delphi method. The complete list of free response suggestions from panel members in Round 1 can be accessed in Additional File 4.

**Table 2:** Round 1 e-Delphi results

	*N*(%)				
	Very important	Important	Not important	Not at all important	I-CVI^a^
PHF Features					
Topographical Parts	46 (73%)	17 (27%)	0 (0%)	0 (0%)	63 (100%)
Displacement of Parts	42 (67%)	21 (33%)	0 (0%)	0 (0%)	63 (100%)
Dislocation	48 (76%)	14 (22%)	1 (2%)	0 (0%)	62 (98%)
Head Shaft Angulation	9 (14%)	43 (68%)	10 (16%)	1 (2%)	52 (83%)
Head Shaft Translation	8 (13%)	43 (68%)	12 (19%)	0 (0%)	51 (81%)
Head Split Fracture	50 (79%)	12 (19%)	1 (2%)	0 (0%)	62 (98%)
Head Impaction	9 (14%)	41 (65%)	12 (19%)	1 (2%)	50 (79%)
Metaphyseal or Calcar Comminution	18 (29%)	31 (49%)	14 (22%)	0 (0%)	49 (78%)
Metaphyseal Head Extension	15 (24%)	35 (56%)	12 (19%)	1 (2%)	50 (79%)
Medial Hinge Disruption	13 (21%)	34 (54%)	16 (25%)	0 (0.0%)	47 (75%)

^a^Consensus was calculated with a content validity index (I-CVI) [78] as the percentage of individuals in agreement on the importance of a PHF feature

### Round 2

In Round 2, surgeons assessed the importance of the 10 original PHF features from Round 1 and the two new features recommended by panel members for the e-Delphi: bone quality and humeral head subluxation. The results of Round 2 can be found in Table 3. Consistent with the results of Round 1, at least 78% of surgeons agreed that dislocation (100%), head split fracture (100%), topographical parts (97%), head shaft translation (93%), displacement of parts (90%), head shaft angulation (83%), and head impaction (83%) are important to clinical decision-making. Bone quality (83%), one of the new additions to Round 2, also met the consensus threshold.

With notably lower agreement, medial hinge disruption (69%) did not meet final consensus. In contrast to the results of Round 1, medial metaphyseal head extension (66%) and calcar comminution (66%) did not meet consensus. Humeral head subluxation (34%) also did not meet consensus. Upon reviewing Round 2 feedback, no new radiographic features were recommended for inclusion in the e-Delphi. Based on these results and the original Delphi recommendations on number of rounds [68] the decision was made by the research team to end the e-Delphi method following Round 2.The list of final PHF features reaching consensus, their definitions, and feature examples are shown in Table 4.

**Table 3:** Round 2 e-Delphi results

	*N*(%)				
	Very important	Important	Not important	Not at all important	I-CVI^a^
PHF Features					
Topographical Parts	24 (83%)	4 (14%)	1 (3%)	0 (0%)	28 (97%)
Displacement of Parts	20 (69%)	6 (21%)	3 (10%)	0 (0%)	26 (90%)
Dislocation	29 (100%)	0 (0%)	0 (0%)	0 (0%)	29 (100%)
Head Shaft Angulation	3 (10%)	21 (72%)	5 (17%)	0 (0%)	24 (83%)
Head Shaft Translation	6 (21%)	21 (72%)	2 (7%)	0 (0%)	27 (93%)
Head Split Fracture	26 (90%)	3 (10%)	0 (0%)	0 (0%)	29 (100%)
Head Impaction	5 (17%)	19 (66%)	5 (17%)	0 (0%)	24 (83%)
Metaphyseal or Calcar Comminution	5 (17%)	14 (48%)	10 (35%)	0 (0%)	19 (66%)
Metaphyseal Head Extension	3 (10%)	16 (55%)	10 (35%)	0 (0%)	19 (66%)
Medial Hinge Disruption	1 (3%)	19 (66%)	9 (31%)	0 (0%)	20 (69%)
Bone Quality	10 (35%)	14 (48%)	5 (17%)	0 (0%)	24 (83%)
Humeral Head Subluxation	3 (10%)	7 (24%)	16 (55%)	3 (10%)	10 (34%)

**Table 4:** Final list of important PHF Features, Definitions, and examples

Fracture Feature	Feature Definition	Feature Examples
Topographical Parts	The location of topographical parts or segments involved in the fracture.	• Head (fracture line at the anatomic neck) • Shaft (fracture line at the surgical neck) • Greater tuberosity • Lesser tuberosity
Displacement of Parts	Greater than 1 cm distance between fragments (or 5 mm for tuberosities) or 45 degrees of angulation between fragments.	• Displaced greater tuberosity fracture • Displaced lesser tuberosity fracture • Displaced surgical neck fracture • Basis for Neer’s 2-part, 3-part and 4-part fracture classification system
Dislocation	The humeral head is not located within the glenoid cavity or socket.	• Anterior or posterior dislocation of the glenohumeral head fragment. • Inferior dislocation
Head Shaft Angulation	The head shaft angle is the angle between the humeral shaft axis and the humeral head axis as observed from the anterior-posterior view.	• Neutral and varus head shaft angulation of < = 140 degrees • Valgus head shaft angulation of > 140 degrees
Head Shaft Translation	The amount of mediolateral displacement between the shaft and the head.	• 44% mediolateral displacement of the head relative to the overall diameter of the metaphysis • Translation > 50% or < = 50% used as a benchmark • Complete translation > 100%
Head Split Fracture	When the articular surface area of the humeral head cleaves into two or more parts.	• May involve at least 20% of the articular surface. • Articular surface is fragmented into a number of separated pieces.
Head Impaction	Forced contact between the head and shaft resulting in impaction and a crushing head deformity.	• At least 50% of the shaft is in contact with the humeral head and has penetrated into the porous bone of the head.
Bone Quality	The bone density of the humerus.	• Could be assessed through the following methods: DXA or DEXA Scan, or the Deltoid Tuberosity Index (DTI), Average Cortical Bone Thickness (CBT), or Tingart measurement on AP X-Rays. • Presence of osteopenia or osteoporosis

^a^Consensus was calculated with a content validity index (I-CVI) [78] as the percentage of individuals in agreement on the importance of a PHF feature

## Discussion

The complexity of the fracture remains an important driver of initial treatment choice for PHF [3]. This study used an e-Delphi method to obtain consensus on the comprehensive list of radiographic fracture features relevant to provider clinical decision-making for PHF. Consistent with prior research [72], we identified topographical parts, displacement of parts, dislocation, and head split fracture, as important features guiding management decisions for PHF. Additionally, our results distinguished head shaft angulation, head shaft translation, head impaction, and bone quality as key radiographic features that are relevant when making treatment decisions for PHF.

The list of features we explored in our e-Delphi was generated from a comprehensive literature review of PHF classification systems; therefore, all the features had some level of previous expert opinion or clinical evidence on their relative value. Yet, with these classification systems spanning between 1960 and 2022, we sought to understand how the features identified in the literature are currently recognized and accepted among experts and how perspectives may have changed with evolving evidence. We began with the most widely known characteristics of a PHF, which are the location and number of parts. The greater tuberosity, lesser tuberosity, anatomic neck, and surgical neck are the four topographical parts of the humerus historically recognized by Neer [42]. The number of parts involved is defined by the presence of displacement, or 1 cm of separation between parts (or 5 mm for the tuberosities) or 45 degrees angulation [42]. Topographical parts and displacement represent two of the most well-known features of PHF [42, 44, 52]. Therefore, not surprisingly, consensus was obtained for topographical parts and displacement in our e-Delphi method.

Dislocation of the humeral head in an anterior or posterior direction can result in persistent pain, nerve palsy, and vascular compromise leading to osteonecrosis, and necessitating more invasive treatment [36, 49, 79]. Dislocation was unanimously identified as “Very Important” by all 29 respondents in Round 2 of our e-Delphi method. Furthermore, all participants in the final round of our e-Delphi method agreed that a head split feature is important for PHF management. Head split fractures are commonly treated operatively due to concerns with avascular necrosis [50], but even when treated operatively, head split fractures have been associated with higher rates of complications, malreduction, and malunion following operative treatment [81, 82].

Our study identified 4 additional fracture characteristics of importance. Head shaft angulation and head shaft translation are two distinct fracture features that can occur due to the position of the head of the humerus relative to the shaft. Head shaft angulation is typically classified as varus (≤ 140 degrees) or valgus (>140 degrees). Head shaft translation, also referred to as shaft displacement or surgical neck displacement in the literature [42, 85, 86], is the amount of mediolateral displacement between the shaft and the head. Evidence suggests that surgical treatment may be preferred in situations where mediolateral translation of the head exceeds 50% of the shaft diameter [61, 87]. Garg et al., identified a significant relationship between increased head shaft translation and poor clinical outcomes [88] and medial head shaft translation of at least 40% has been associated with significantly greater odds of nerve damage [86]. Both head shaft angulation of 90 degrees or less and head shaft translation of at least 50% have also been implicated in PHF nonunion following conservative treatment [37]. Our e-Delphi method is the first to identify consensus for head shaft angulation and head shaft translation for PHF.

Our e-Delphi method also identified the importance of head impaction for PHF management. Conservative treatment for impacted head fractures with minimal displacement is effective [89], but surgical treatment may be preferable in more complex PHF with greater displacement [90]. Low DASH and ASES scores have been observed in impacted fractures treated conservatively where the head is angulated and impacted [91]. Lastly, bone quality was recommended by multiple surgeons in Round 1 for inclusion as a PHF feature in the e-Delphi and met consensus as an important PHF feature in our e-Delphi method in Round 2. Poor bone quality is more common in the elderly and has been associated with a higher degree of surgical complications, specifically a higher likelihood of screw perforation into the articular space following ORIF [94]. Bone quality is recognized as an important feature, but has not previously been identified in a Delphi method.

E-Delphi approaches have recently been used to explore PHF features and decision making. Two separate studies recently ranked key patient and fracture characteristics influencing treatment decisions of PHFs [72] and attempted to determine treatment consensus on combined patient and fracture types [73]. Klute, et al., (2023) conducted a ranked e-Delphi method to assess patient and clinical characteristics relevant for PHF decision-making among a group of 18 orthopaedic shoulder and trauma surgeons from Austria, Germany, and Switzerland [72]. Similar to our work, Klute et al., (2023) reached consensus on dislocation, head split, and displacement of the tuberosities (included within our displacement/topographical parts) as important features guiding management decisions for PHF. Together, our findings indicate the collective importance of dislocation, head split fractures, and displacement of the tuberosities on treatment decision-making for PHF. However, angulation did not reach consensus in Klute’s work and there was not overlap in the remaining characteristics. Some differences observed between studies may be due to differences in the regional perspectives among American and international surgeons. Though, it’s also likely that the ranked methodology restricted the full list of fracture characteristics which may have been selected by participants.

Recently, Williams et al., (2025) attempted to identify treatment consensus across seven fracture patterns while varying patients age and health characteristics [73]. A vignette approach was used to present common patient and fracture subtypes. Among those presented, treatment consensus was only reached for 8 of the 14 fracture and patient types, signaling little consensus exists on operative or non-operative treatment for common fracture presentations. Furthermore, this work only represented 7 fracture patterns, and somewhat narrow patient characteristics. The challenge with this approach is the number of vignettes that would need to be presented to comprehensively span the diversity of patient and fracture patterns that are presented in real-world practice. The methodology used by Williams et al. was quite different than ours, but fracture characteristics which were included in both our work and Williams et al. included part location and displacement, head shaft angulation, head shaft translation, and dislocation. The inclusion of these characteristics may support their recognized importance in management decisions. In summary, although there was some consistency in characteristics used or selection across our work and that of Klute [72] and Williams [73], our approach was distinct in that we sought to obtain a comprehensive list of all radiographic PHF features important to clinical decisions.

It is important to note the limitations of this study. While the focus of this work was identifying the comprehensive list of PHF features most relevant to treatment decision-making, we emphasize that fracture features should never be the sole deciding factor in the treatment a patient receives. In addition, it is important to acknowledge that while the Delphi method is an established consensus methodology [68], these results are limited to the expert opinion of the respondents. Our consensus was formed based on a group of 29 orthopaedic surgeons practicing primarily across the United States, which might reflect regional or national perspectives unique to the US. Though this group size is larger than used by similar work [72], and exceeds the general Delphi methodology standard of 12 participants needed to enable consensus [74, 75]. Additionally, it is important to note that although the majority of our respondents were located in the US, some of our results were consistent with Klute’s panel of experts selected from Austria, Germany and Switzerland which signals multi-national consensus on feature importance for the case of tuberosity displacement, dislocation and head split fractures.

## Conclusions

In this study we identify a list of 8 important PHF radiographic features that reached consensus from orthopaedic surgeons following an e-Delphi method. A next step in advancing care for patients with PHF are large observational studies that measure the range of complexity of both fracture and patient characteristics in the real-world, facilitating the study of complex PHF fracture patterns and patient outcomes. A comprehensive understanding and measurement of all important fracture-specific and patient-specific factors will allow us to generate evidence to improve individual treatment decision making for PHF.

## Supplementary Information

Below is the link to the electronic supplementary material.

Supplementary Material 1 - Delphi Invitation

Supplementary Material 2 - Round 1

Supplementary Material 3 - Round 1 Feedback

Supplementary Material 4 - Round 2

## Data Availability

The datasets used and/or analyzed during the current study are available from the corresponding author on reasonable request.

## Abbreviations

PHF: Proximal humerus fracture
CINAHL: Cumulative Index to Nursing and Allied Health Literature
OSWG: Orthopaedic Surgeon Working Group
I-CVI: Item-level content validity index

## Declarations

### e-Delphi panel members

Adam Hines, Legacy Orthopedics & Sports Medicine, TX, United States.

Andrew Bries, ORA Orthopedics, IA, United States.

Benjamin Cooper, Sauk Prairie Healthcare, WI, United States.

Brad Winter, OrthoCarolina, NC, United States.

Brandon Rogalski, Medical University of South Carolina, SC, United States.

Brent Norris, Oklahoma State University, OK, United States.

Brian Davis, BoulderCentre for Orthopedics & Spine, CO, United States.

Chris Boullion, Grand Strand Medical Center, SC, United States.

Colin Eakin, Palo Alto Medical Foundation, CA, United States.

Curtis Noel, Crystal Clinic Orthopaedic Center, OH, United States.

Daniel Cunningham, Prisma Health - Midlands, SC, United States.

Daryl Dillman, Spartanburg Regional Healthcare System, SC, United States.

David Weiss, UVA Health, VA, United States.

Derik Geist, West Virginia University, WV, United States.

Dickson Schaefer, Cape Fear Valley Medical Center, NC, United States.

Duncan Mackinlay, Bluewater Health, Ontario, Canada.

Erin Hofer, Tristar Skyline Medical Center, TN, United States.

Grant Bennett, OrthoArkansas, AR, United States.

Greg Colbath, Spartanburg Regional Healthcare System, SC, United States.

J. Field Scovell, Carrell Clinic, TX, United States.

James Spearman, Prisma Health - Upstate, SC, United States.

Jarrod Dumpe, Atrium Health Navicent, GA, United States.

Jeff Abrams, Penn Medicine Princeton Medical Center, NJ, United States.

Jenny Bruggers, Wellstar Kennestone Regional Medical Center, GA, United States.

Jessica Chiang, Charleston Area Medical Center, WV, United States.

John Christoforetti, The Centers for Advanced Orthopedics, MD, United States.

John Moor, John T Moor MD, PA, FL, United States.

John Stefancin, Southwoods Health, OH, United States.

Justin Kauk, WakeMed Orthopedics, NC, United States.

Justin T. Smith, Atrium Health, NC, United States.

Kevin Murr, Prisma Health - Midlands, SC, United States.

Kristoff Reid, Medical University of South Carolina, SC, United States.

Kurt Yusi, Bon Secours St. Francis, SC, United States.

Kyle Watford, Bon Secours St. Francis, SC, United States.

Langdon Hartsock, Medical University of South Carolina, SC, United States.

Lisa Cannada, Novant Health, NC, United States.

Louis C. Grandizio, Geisinger Health System, PA, United States.

Lucas Buchler, Northwestern Medicine, IL, United States.

Lucas Teske, Tennessee Orthopaedic Alliance, TN, United States.

Madhav Karunakar, Atrium Health, NC, United States.

Mara Schenker, Grady Memorial Hospital, GA, United States.

Matthew T. Nugent, Oregon Shoulder Institute, OR, United States.

Mike Milne, Motion Orthopaedics, MO, United States.

Misty Suri, Ochsner Health System, LA, United States.

Richard Borgatti, Seaview Orthopaedics, NJ, United States.

Richard Hawkins, Greenville Health System, SC, United States.

Rick Angelo, Evergreen Medical Center, WA, United States.

Robert Satterfield, Emerge Ortho - Wilson Division, NC, United States.

Russell Norris, ECU Health, NC, United States.

Ryan Blackwell, Orthopedic ONE, OH, United States.

Scott Broderick, Spartanburg Regional Healthcare System, SC, United States.

Seth Yarboro, UVA Health, VA, United States.

Shawn Yeazell, ECU Health, NC, United States.

Spero Karas, Emory Healthcare, GA, United States.

Stefan J. Tolan, Steadman Hawkins Clinic of the Carolinas; Prisma Health - Upstate, SC, United States.

Stephen Hamilton, Beacon Orthopedics & Sports Medicine, OH, United States.

Stephen Sierra, Acension Providence, MI, United States.

Suman Medda, Atrium Health Carolinas Medical Center, NC, United States.

Thomas Alexander, Optim Orthopedics, GA, United States.

Thomas Noonan, University of Colorado Medicine, CO, United States.

Timothy O’Brien, Alpine Orthopedics, MT, United States.

Todd Michener, Chester County Hospital, PA, United States.

William Obremskey, Vanderbilt Health, TN, United States.
