# Supplementary material for: Delphi method consensus on radiographic characteristics influencing management decisions for proximal humerus fracture
Source: J Orthop Surg Res. 2025 Nov 26;20:1041. doi: 10.1186/s13018-025-06465-w (PMC12659269; doi:10.1186/s13018-025-06465-w)
Supplement: Supplementary file 1 — Supplementary Material 1 - Delphi Invitation [file 13018_2025_6465_MOESM1_ESM.pdf]

A Delphi Process to Gain Consensus on the Important Features for Classification of Proximal Humerus Fractures

Dear colleague,

We are conducting a Delphi process to obtain consensus on the most important fracture features for the diagnosis and classification of proximal humerus fractures (PHF). In this NIH-funded study, we will develop a deep learning model to analyze shoulder X-rays and identify and report fracture features. The long-term goal is that this information would be readily available for physicians in the EHR after a shoulder X-ray is taken. We believe this project has the potential to improve the accuracy of diagnosis, improve the efficiency of care for PHF, and ultimately improve patient outcomes.

We are looking for orthopaedic surgeons with experience treating PHF who would be interested in participating in our Delphi process. We aim to recruit an international panel of 100 orthopaedic surgeons to participate. Participants will be asked to complete 3 online surveys, each taking between 10-15 minutes to complete. There is no risk, compensation, or direct benefit to participating in the Delphi process, but your input may

provide beneficial information to improve patient care. Your information will be kept confidential. Individual names and responses will not be reported, but all participants will receive acknowledgment in publications with a group name.

Preliminary Results in Model Accuracy Identifying the Neer Classification

To date we have built preliminary deep learning models to classify fractures using the Neer classification, and have had success identifying 1-part, 2-part, 3-part and 4-part fractures. Our current deep learning model is built on a series of approximately 400 images and has accuracy as high at 97% in correctly identifying 1-part fractures. We plan to improve this model's accuracy by increasing our sample size and expanding upon the fracture features identified.

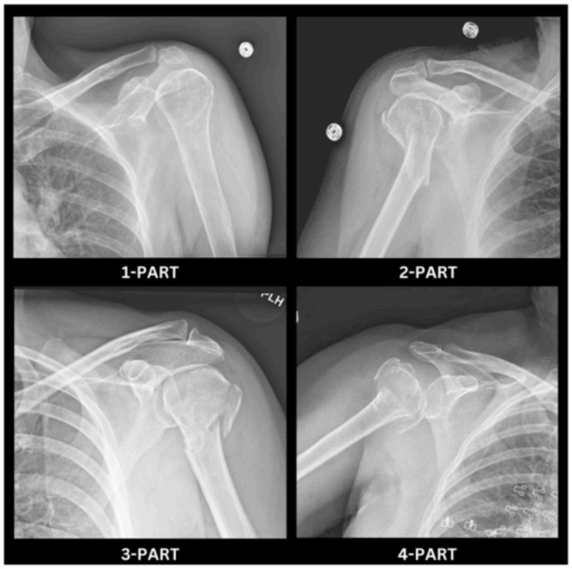

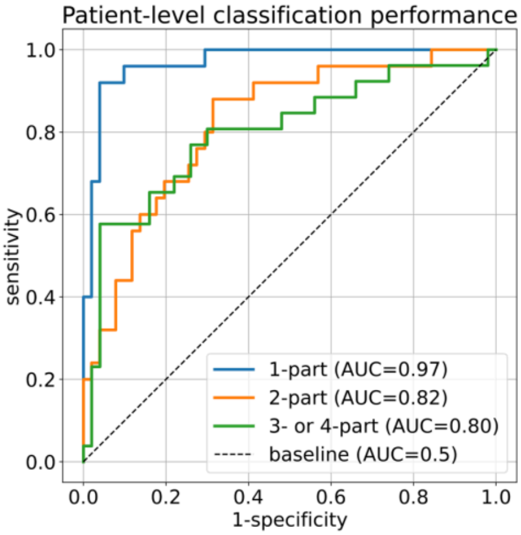

We strongly believe this project will improve efficiency and outcomes for patients with PHF. We hope you will consider participating in this important process. Please complete the following survey questions if you are interested in participating.

Thank you,

The Orthopaedic Surgeon Working Group

Are you interested in participating in the Delphi process?

- ◯ Yes
- ◯ No

Personal Information

First and Last Name

Gender

- ◯ Male

○ Female

○ Other

Email

[ ]

Where do you currently practice?

○ Academic medical center

○ Private practice

Name of hospital/institution or practice

[ ]

Professional Experience

What is your subspecialty? (if any)

- ◯ General Orthopaedics
- ◯ Orthopaedic Trauma
- ◯ Orthopaedic Shoulder Surgeon
- ◯ Sports Medicine
- ◯ Hand
- ◯ [ ] Other

Advanced degree(s)

- ☐ MD
- ☐ DO
- ☐ PhD
- ☐ MPH
- ☐ MBA
- ☐ MSc
- ☐ Other

What is your current level of experience in orthopaedic surgery?

- ◯ Residency
- ◯ Fellowship

- First year of independent practice
- 2-5 years of practice
- 6-10 years of practice
- 11-15 years of practice
- More than 15 years of practice

On average, how many proximal humerus fractures do you treat in a typical year?

- 0-20
- 21-50
- 50-100
- More than 100

Powered by Qualtrics
