# Supplementary material for: Delphi method consensus on radiographic characteristics influencing management decisions for proximal humerus fracture
Source: J Orthop Surg Res. 2025 Nov 26;20:1041. doi: 10.1186/s13018-025-06465-w (PMC12659269; doi:10.1186/s13018-025-06465-w)
Supplement: Supplementary file 2 — Supplementary Material 2 - Round 1 [file 13018_2025_6465_MOESM2_ESM.pdf]

A Delphi Method to Gain Consensus on Important Proximal Humerus Fracture Features

Thank you for your participation in this NIH-funded study. Our goal is to obtain consensus on the proximal humerus fracture (PHF) features that affect treatment effectiveness and outcomes. Using the features agreed upon through this survey, we will develop an Artificial Intelligence model to read, identify, and output important fracture features from X-rays. We believe this project has the potential to improve the accuracy of diagnosis, improve the efficiency of care for PHF, and ultimately improve patient outcomes.

There will be up to 3 rounds of surveys which will be sent to you via email. We anticipate that each survey will take 5-10 minutes to complete. There is no risk, compensation, or direct benefit to participating in the Delphi process, but your input may provide beneficial information to improve patient care. Your information will be kept confidential as individual names and responses will not be reported, but all participants will receive acknowledgment in publications with a group name. Your responses to these questions will serve as agreement to participate in this study.

Please provide your email so we can track your responses across survey rounds.

Round 1 - Identifying Important Fracture Features

We will provide you with a general fracture feature definition and a range of examples for each feature found in the literature. Because some fracture features lack a consensus definition, there will be a range in some feature examples. However, we ask that you evaluate the feature concept importance on PHF treatment effectiveness and outcomes. You will be given the opportunity to offer a revised feature definition if you'd like.

When evaluating each feature, please imagine you are looking at an acute PHF X-ray image or series of images taken within 2 weeks of the injury date. *Please evaluate how important each feature would be to your treatment decision-making.*

TOPOGRAPHICAL PARTS

Definition: The location of topographical parts or segments involved in the fracture.

Feature Examples:

- Head (fracture line at the anatomic neck)
- Shaft (fracture line at the surgical neck)
- Greater tuberosity
- Lesser tuberosity

How important are the topographical parts involved in the fracture when making treatment decisions for PHF?

- ○ Very important
- ○ Important
- ○ Not important
- ○ Not at all important

Optional: Please provide an alternative **topographical parts** feature definition or example.

DISPLACEMENT OF PARTS

Definition: Greater than 1 cm distance between fragments (or 5 mm for tuberosities) or 45 degrees of angulation between fragments.

Feature Examples:

- Displaced greater tuberosity fracture
- Displaced surgical neck fracture
- Basis for Neer's 2-part, 3-part and 4-part fracture classification system

How important is displacement of parts when making treatment decisions for PHF?

- ○ Very important
- ○ Important
- ○ Not important
- ○ Not at all important

Optional: Please provide an alternative **displacement of parts** feature definition or example.

DISLOCATION

Definition: The humeral head is not located within the glenoid cavity or socket.

Feature Examples:

- Anterior or posterior dislocation of the glenohumeral head fragment.
- Inferior dislocation

How important is dislocation when making treatment decisions for PHF?

○ Very important
○ Important
○ Not important
○ Not at all important

Optional: Please provide an alternative **dislocation** feature definition or example.

HEAD-SHAFT ANGULATION

Definition: The head-shaft angle is the angle between the humeral shaft axis and the humeral head axis as observed from the anterior-posterior view.

Feature Examples:

- Neutral and varus head shaft angulation of $<= 140$ degrees
- Valgus head shaft angulation of > 140 degrees

How important is head-shaft angulation when making treatment decisions for PHF?

- ○ Very important
- ○ Important
- ○ Not important
- ○ Not at all important

Optional: Please provide an alternative **head-shaft angulation** feature definition or example.

HEAD-SHAFT TRANSLATION

Definition: The amount of mediolateral displacement between the shaft and the head.

Feature Examples:

- 44% mediolateral displacement of the head relative to the overall diameter of the metaphysis

- Translation > 50% or <= 50% used as a benchmark

How important is head-shaft translation when making treatment decisions for PHF?

- ○ Very important
- ○ Important
- ○ Not important
- ○ Not at all important

Optional: Please provide an alternative **head-shaft translation** feature definition or example.

[ ]

HEAD SPLIT FRACTURE

Definition: When the articular surface area of the humeral head cleaves into two or more parts.

Feature Examples:

- May involve at least 20% of the articular surface.
- Articular surface is fragmented into a number of separated pieces.

How important is the presence of a head split fracture when making treatment decisions for PHF?

- ◯ Very important
- ◯ Important
- ◯ Not important
- ◯ Not at all important

Optional: Please provide an alternative **head split** feature definition or example.

HEAD IMPACTION

Definition: Forced contact between the head and shaft resulting in impaction and a crushing head deformity.

Feature Examples:

- At least 50% of the shaft is in contact with the humeral head and has penetrated into the porous bone of the head.

How important is the presence of head impaction when making treatment

decisions for PHF?

- ○ Very important
- ○ Important
- ○ Not important
- ○ Not at all important

Optional: Please provide an alternative **head impaction** feature definition or example.

METAPHYSEAL OR CALCAR COMMINUTION

Definition: The presence of one or more intermediate fragments in the medial calcar area.

Feature Examples:

- Degree of comminution may be measured by the number of fragments separated from the humerus in the calcar area.

How important is metaphyseal or calcar comminution when making treatment decisions for PHF?

- ○ Very important
- ○ Important
- ○ Not important
- ○ Not at all important

Optional: Please provide an alternative **metaphyseal or calcar comminution** feature definition or example.

METAPHYSEAL HEAD EXTENSION

Definition: The length of the medial calcar segment that remains attached to the head.

Feature Examples:

- If < 8mm then calcar disruption is present.
- If >= 8mm then the calcar is intact.

How important is metaphyseal head extension when making treatment decisions for PHF?

- ○ Very important
- ○ Important

○ Not important
○ Not at all important

Optional: Please provide an alternative **metaphyseal head extension** feature definition or example.

[ ]

MEDIAL HINGE DISRUPTION

Definition: Separation of the shaft and head causing disruption of the pivot point of the head at the level of the posteromedial fracture line.

Feature Examples:

- Separation of the shaft and head at the calcar region by > 2 mm (hinge disruption)

How important is medial hinge disruption when making treatment decisions for PHF?

○ Very important
○ Important
○ Not important

○ Not at all important

Optional: Please provide an alternative **medial hinge disruption** feature definition or example.

Are there any fracture features you believe to be important that are missing?

○ Yes
○ No

Please list the missing fracture features.

Powered by Qualtrics
