# Supplementary material for: Delphi method consensus on radiographic characteristics influencing management decisions for proximal humerus fracture
Source: J Orthop Surg Res. 2025 Nov 26;20:1041. doi: 10.1186/s13018-025-06465-w (PMC12659269; doi:10.1186/s13018-025-06465-w)
Supplement: Supplementary file 3 — Supplementary Material 3 - Round 1 Feedback [file 13018_2025_6465_MOESM3_ESM.docx]

**Round 1 Feedback**

Topographical Parts

“Bone Quality”

“Varus impaction/ medial calcar compromise”

“Displacement magnitude”

“Head split”

“Sections metaphase, etc”

“bicipital groove”

“Displacement”

Displacement of Parts

“Displacement of the lesser tuber”

“complete displacement or incomplete (100%) shaft relative to articular surface”

“Angulation”

“Superior or posterior displacement”

“Rotation of fracture fragment”

“Direction of displacement proximal vs posterior”

“Measurements angulation, bone loss”

Dislocation

“Subluxation”

“Inferior subluxation can occur without true dislocation”

“Head split”

“Angulation of fragments”

Head Shaft Angulation

“Comminution”

“This should be true AP view to the glenohumeral joint (Grashey view), not perpendicular to the chest (AP view); Should also measure head-shaft angulation on Scapular Y view”

“Angulation plus displacement”

“Rotation”

Head Shaft Translation

“greater or less than 100%”

“Shortening”

“This is most relevant if measured on upright XR with patient holding arm in neutral, supported position (where it will heal)”

“Percentage of contact”

Head Split Fracture

“Depression depth”

“Percentage of intact head”

Head Impaction

“Loose bodies”

“Important to distinguish between impaction of shaft and other fragments (i.e. tuberosities”

“Surface area”

Calcar Comminution

“Maintenance of Gothic arch”

“Stability and bone loss”

Metaphyseal Head Extension

“Important to distinguish between calcar (medial metaphysis), and metaphysis.”

“Inferior extension”

Medial Hinge Disruption

“Angulation”

“Unstable bone loss and comminution”

Missing features

“Complete displacement (>100%) medial displacement of surgical neck fractures”

“Cuff insertion site, impingement”

“Osteopenia bone quality or modified Dorr classification”

“cortical thickness, pseudo subluxation - axillary nerve damage, is xray upright or supine, arrow for upright”

“bone density, osteopenia, patient age, arm dominance, activity level”

“Level of arthritic changes”

“bone quality, pre OA”

“Bone quality”

“bone quality”

“Comminution or segmental fracture of the proximal shaft between surgical neck and deltoid tuberosity”

“Pre existing OA, RCT arthropathy”

“bone quality, cortical thickness”

“Bone quality”

“Combined patterns ie head split dislocation”

“Bone quality”

“bone quality”

“displacement of the tuberosities, age, pre shoulder OA, thickness of the head segment that remains”
