# Supplementary material for: Delphi method consensus on radiographic characteristics influencing management decisions for proximal humerus fracture
Source: J Orthop Surg Res. 2025 Nov 26;20:1041. doi: 10.1186/s13018-025-06465-w (PMC12659269; doi:10.1186/s13018-025-06465-w)
Supplement: Supplementary file 4 — Supplementary Material 4 - Round 2 [file 13018_2025_6465_MOESM4_ESM.pdf]

A Delphi Method to Gain Consensus on Important Proximal Humerus Fracture Features - Round 2

In line with the Delphi methodology, in the second round **we will show you the aggregate results from Round 1**, and ask you to **assess the importance of the original list of fracture features as well as the importance of new features suggested from panel participants in Round 1**.

There is no risk, compensation, or direct benefit to participating in the Delphi method, but your input may provide beneficial information to improve patient care. Your information will be kept confidential as individual names and responses will not be reported, but all participants will receive acknowledgment in publications with a group name. Your responses to these questions will serve as agreement to participate in this study.

Please provide your email so we can track your responses across survey rounds.

Round 2 - Obtaining Consensus on Important Fracture Features

In this round, you will first review the aggregate results from Round 1 on the importance of each PHF fracture feature.

Please see the results from Round 1 below:

Round 1 Results				
	N(%)			
	Very important	Important	Not important	Not at all important
**PHF Features**				
Topographical Parts	46 (73%)	17 (27%)	0 (0%)	0 (0%)
Displacement of Parts	42 (67%)	21 (33%)	0 (0%)	0 (0%)
Dislocation	48 (76%)	14 (22%)	1 (1.6%)	0 (0%)
Head Shaft Angulation	9 (14%)	43 (68%)	10 (16%)	1 (1.6%)
Head Shaft Translation	8 (13%)	43 (68%)	12 (19%)	0 (0%)
Head Split Fracture	50 (79%)	12 (19%)	1 (1.6%)	0 (0%)
Head Impaction	9 (14%)	41 (65%)	12 (19%)	1 (1.6%)
Metaphyseal or Calcar Comminution	18 (29%)	31 (49%)	14 (22%)	0 (0%)
Metaphyseal Head Extension	15 (24%)	35 (56%)	12 (19%)	1 (1.6%)
Medial Hinge Disruption	13 (21%)	34 (54%)	16 (25%)	0 (0%)

Similar to Round 1, we will provide you with a general fracture feature definition and a range of examples for each feature found in the literature. The *original fracture features from Round 1* and *new radiographic fracture features that were recommended by participants in Round 1* are included for your evaluation in this round. Any modifications to the original fracture features from participant suggestions in Round 1 are shown in red.

When evaluating each feature, please imagine you are looking at an acute PHF X-ray image or series of images taken within 2 weeks of the injury date. *Please evaluate how important each feature would be to your treatment decision-making.*

TOPOGRAPHICAL PARTS

Definition: The location of topographical parts or segments involved in the fracture.

Feature Examples:

- Head (fracture line at the anatomic neck)
- Shaft (fracture line at the surgical neck)
- Greater tuberosity
- Lesser tuberosity

How important are the topographical parts involved in the fracture when making treatment decisions for PHF?

- ○ Very important
- ○ Important
- ○ Not important
- ○ Not at all important

Optional: Please provide an alternative **topographical parts** feature definition or example.

[ ]

DISPLACEMENT OF PARTS

Definition: Greater than 1 cm distance between fragments (or 5 mm for tuberosities) or 45 degrees of angulation between fragments.

Feature Examples:

- Displaced greater tuberosity fracture
- Displaced lesser tuberosity fracture

- Displaced surgical neck fracture
- Basis for Neer's 2-part, 3-part and 4-part fracture classification system

How important is displacement of parts when making treatment decisions for PHF?

- ○ Very important
- ○ Important
- ○ Not important
- ○ Not at all important

Optional: Please provide an alternative **displacement of parts** feature definition or example.

DISLOCATION

Definition: The humeral head is not located within the glenoid cavity or socket.

Feature Examples:

- Anterior or posterior dislocation of the glenohumeral head fragment.
- Inferior dislocation

How important is dislocation when making treatment decisions for PHF?

- ◯ Very important
- ◯ Important
- ◯ Not important
- ◯ Not at all important

Optional: Please provide an alternative **dislocation** feature definition or example.

HEAD-SHAFT ANGULATION

Definition: The head-shaft angle is the angle between the humeral shaft axis and the humeral head axis as observed from the anterior-posterior view.

Feature Examples:

- Neutral and varus head shaft angulation of <= 140 degrees
- Valgus head shaft angulation of > 140 degrees

How important is head-shaft angulation when making treatment decisions for PHF?

- ○ Very important
- ○ Important
- ○ Not important
- ○ Not at all important

Optional: Please provide an alternative **head-shaft angulation** feature definition or example.

[ ]

HEAD-SHAFT TRANSLATION

Definition: The amount of mediolateral displacement between the shaft and the head.

Feature Examples:

- 44% mediolateral displacement of the head relative to the overall diameter of the metaphysis
- Translation > 50% or <= 50% used as a benchmark
- Complete translation > 100%

How important is head-shaft translation when making treatment decisions for PHF?

- ○ Very important
- ○ Important
- ○ Not important
- ○ Not at all important

Optional: Please provide an alternative **head-shaft translation** feature definition or example.

HEAD SPLIT FRACTURE

Definition: When the articular surface area of the humeral head cleaves into two or more parts.

Feature Examples:

- May involve at least 20% of the articular surface.
- Articular surface is fragmented into a number of separated pieces.

How important is the presence of a head split fracture when making treatment decisions for PHF?

- ◯ Very important
- ◯ Important
- ◯ Not important
- ◯ Not at all important

Optional: Please provide an alternative **head split** feature definition or example.

HEAD IMPACTION

Definition: Forced contact between the head and shaft resulting in impaction and a crushing head deformity.

Feature Examples:

- At least 50% of the shaft is in contact with the humeral head and has penetrated into the porous bone of the head.

How important is the presence of head impaction when making treatment

decisions for PHF?

- ◯ Very important
- ◯ Important
- ◯ Not important
- ◯ Not at all important

Optional: Please provide an alternative **head impaction** feature definition or example.

METAPHYSEAL OR CALCAR COMMINUTION

Definition: The presence of one or more intermediate fragments in the medial calcar area.

Feature Examples:

- Degree of comminution may be measured by the number of fragments separated from the humerus in the calcar area.

How important is metaphyseal or calcar comminution when making treatment decisions for PHF?

○ Very important
○ Important
○ Not important
○ Not at all important

Optional: Please provide an alternative **metaphyseal or calcar comminution** feature definition or example.

MEDIAL METAPHYSEAL HEAD EXTENSION

Definition: The length of the calcar (medial metaphysis) segment that remains attached to the head.

Feature Examples:

- If < 8mm then calcar disruption is present.
- If >= 8mm then the calcar is intact.

How important is medial metaphyseal head extension when making treatment decisions for PHF?

○ Very important

○ Important
○ Not important
○ Not at all important

Optional: Please provide an alternative **medial metaphyseal head extension** feature definition or example.

MEDIAL HINGE DISRUPTION

Definition: Separation of the shaft and head causing disruption of the pivot point of the head at the level of the posteromedial fracture line.

Feature Examples:

- Separation of the shaft and head at the calcar region by > 2 mm (hinge disruption)

How important is medial hinge disruption when making treatment decisions for PHF?

○ Very important
○ Important

◯ Not important

◯ Not at all important

Optional: Please provide an alternative **medial hinge disruption** feature definition or example.

The following fracture features are new additions based on the feedback from participants in Round 1. ***Please evaluate the importance of these features to your treatment decision-making.***

BONE QUALITY

Definition: The bone density of the humerus.

Feature Examples:

- Could be assessed through the following methods: DXA or DEXA Scan, or the Deltoid Tuberosity Index (DTI), Average Cortical Bone Thickness (CBT), or Tingart measurement on AP X-Rays.

- Presence of osteopenia or osteoporosis

How important is bone quality when making treatment decisions for PHF?

- ○ Very important
- ○ Important
- ○ Not important
- ○ Not at all important

Optional: Please provide an alternative **bone quality** feature definition or example.

HUMERAL HEAD SUBLUXATION

Definition: Inferior or posterior partial dislocation of the humeral head from the glenoid.

Feature Examples:

- The midpoint of the head of the humerus shifts below the lower part of the glenoid, violating the gothic arch (Cirino et al., 2022).
- Could be caused by injury to the axillary nerve.

How important is humeral head subluxation when making treatment decisions for PHF?

- ◯ Very important
- ◯ Important
- ◯ Not important
- ◯ Not at all important

Optional: Please provide an alternative **humeral head subluxation** feature definition or example.

Are there any fracture features you believe to be important that are missing?

- ◯ Yes
- ◯ No

Please list the missing fracture features.

Powered by Qualtrics
